# Metformin inhibits Branched Chain Amino Acid (BCAA) derived ketoacidosis and promotes metabolic homeostasis in MSUD

**DOI:** 10.1038/srep28775

**Published:** 2016-07-04

**Authors:** Davis S. Sonnet, Monique N. O’Leary, Mark A. Gutierrez, Steven M. Nguyen, Samiha Mateen, Yuehmei Hsu, Kylie P. Mitchell, Antonio J. Lopez, Jerry Vockley, Brian K. Kennedy, Arvind Ramanathan

**Affiliations:** 1 Buck Institute for Research on Aging, 8001 Redwood Blvd, Novato, CA 94945, USA; 2 University of Michigan, Department of Pathology, 1150 W Medical Center Dr #7520, Ann Arbor, MI 48109, USA; 3 University of Colorado, Denver Anschutz Medical Campus 13001 E. 17th Pl. Aurora, Colorado, 80045, USA; 4 University of California, Davis 1 Shields Ave, Davis, CA 95616, USA; 5 University of Pittsburgh School of Medicine, Department of Pediatrics, Children’s Hospital of Pittsburgh, 4401 Penn Avenue, Pittsburgh, PA 153224.

## Abstract

Maple Syrup Urine Disease (MSUD) is an inherited disorder caused by the dysfunction in the branched chain keto-acid dehydrogenase (BCKDH) enzyme. This leads to buildup of branched-chain keto-acids (BCKA) and branched-chain amino acids (BCAA) in body fluids (e.g. keto-isocaproic acid from the BCAA leucine), leading to numerous clinical features including a less understood skeletal muscle dysfunction in patients. KIC is an inhibitor of mitochondrial function at disease relevant concentrations. A murine model of intermediate MSUD (iMSUD) shows significant skeletal muscle dysfunction as by judged decreased muscle fiber diameter. MSUD is an orphan disease with a need for novel drug interventions. Here using a 96-well plate (liquid chromatography- mass spectrometry (LC-MS) based drug-screening platform we show that Metformin, a widely used anti-diabetic drug, reduces levels of KIC in patient-derived fibroblasts by 20–50%. This Metformin-mediated effect was conserved *in vivo*; Metformin-treatment significantly reduced levels of KIC in the muscle (by 69%) and serum (by 56%) isolated from iMSUD mice, and restored levels of mitochondrial metabolites (e.g. AMP and other TCA). The drug also decreased the expression of mitochondrial branched chain amino transferase (BCAT) which produces KIC in skeletal muscle. This suggests that Metformin can restore skeletal muscle homeostasis in MSUD by decreasing mitochondrial KIC production.

Maple Syrup Urine Disease (MSUD) is an inherited disorder in branched-chain amino acid (BCAA) catabolism caused by mutations in genes that encode the components of the branched chain keto-acid dehydrogenase (BCKDH) complex, which catalyzes the first irreversible step in BCAA catabolism^1^,^2^,^3^. BCKDH dysfunction results in the accumulation of the keto-acids ketoisocaproic acid (KIC) from leucine, ketoisovaleric acid (KIV) from valine and ketomethylvaleric acid (KMV) from isoleucine, produced by the upstream enzymes cytosolic branched-chain amino acid transaminase (BCAT-1) and mitochondrial BCAT-2 (Fig. 1a). BCAT-2 is the major isoform expressed in skeletal muscle, and referred to henceforth as BCAT. MSUD is classified as type I, II or III based on mutations that occur in the E1, E2, or E3 complexes of BCKDH, respectively. The disease was first described by Menkes *et al.* in 1954^4^ as a progressive cerebral dysfunction and in 1960, was recognized as a branched chain ketoaciduria by Dancis *et al.*^2^. In the classic form of MSUD^2^,^5^,^6^, BCKDH enzyme only has 2–5% normal activity. In addition to the classic form, there are intermediate^7^,^8^ (15–25% BCKDH activity), intermittent (asymptomatic until 10–16 months or later) and thiamine-responsive^9^,^10^ MSUD diseases. Thiamine responsivity is associated with a specific mutation in the thiamine binding site in the E1b subunit, or due to the stabilization of BCKDH via an allosteric interaction^11^.

It has been recognized that MSUD patients display skeletal-muscle abnormalities including lesions in myofibers^12^ the underlying mechanism of which is less understood. Skeletal muscle tissue is the major site of amino acid catabolism and a source of Branched Chain Amino acid derived Keto-acids (BCKA). The concentrations of leucine and BCAAs in the blood range from approximately 1 to 5 mM in MSUD patients^13^ (compared to approximately 90–250 μM in unaffected adult subjects^14^,^15^,^16^). BCAA concentrations of up to 1 mM are routinely used in DMEM media for culturing of myotubes, suggesting that accumulated KIC in muscle might also play an important role in mediating skeletal muscle metabolic-dysfunction *in vitro* and *in vivo*. BCKDH and its downstream enzymes provide an alternative source of Succinyl and Acetyl CoA for the TCA cycle, along with glucose (Fig. 1a). In this study we show that BCAA derived keto-acidosis is an important cause of skeletal muscle metabolic and mitochondrial dysfunction, and is a target for alleviating MSUD.

The primary treatment of MSUD in patients is via the dietary restriction of BCAA^17^,^18^. Since leucine, valine and isoleucine are essential amino acids, dietary compliance can therefore be challenging. An alternative approach to significantly improve outcomes is liver transplantation^18^,^19^. This invasive procedure presents its own challenges in terms of organ availability, high costs and other risks. Therefore, a great need exists for new therapeutic approaches, for instance an efficacious small molecule. Here we developed a high throughput LC-MS based small molecule-screening of patient-derived dermal fibroblasts to discover new drugs that can diminish accumulation of the toxic metabolite KIC and alleviate mitochondrial dysfunction associated with MSUD. Using this platform, we show that Metformin, a widely used anti-diabetic drug, diminishes levels of KIC in patient-derived fibroblasts. The effect of Metformin on inhibiting KIC production discovered using patient fibroblasts, was also conserved *in vivo* in an iMSUD mouse model. We also show that intramuscular leucine accumulation is reduced by Metformin. Metformin reprograms the metabolism of MSUD cells by inhibiting BCAA catabolism rescuing the inhibitory effects of KIC on mitochondrial metabolism. This drug provides a readily translatable strategy for managing MSUD and possibly other diseases associated with BCAA catabolism.

## Results

### iMSUD mice exhibit skeletal muscle atrophy

**Figure 1:**
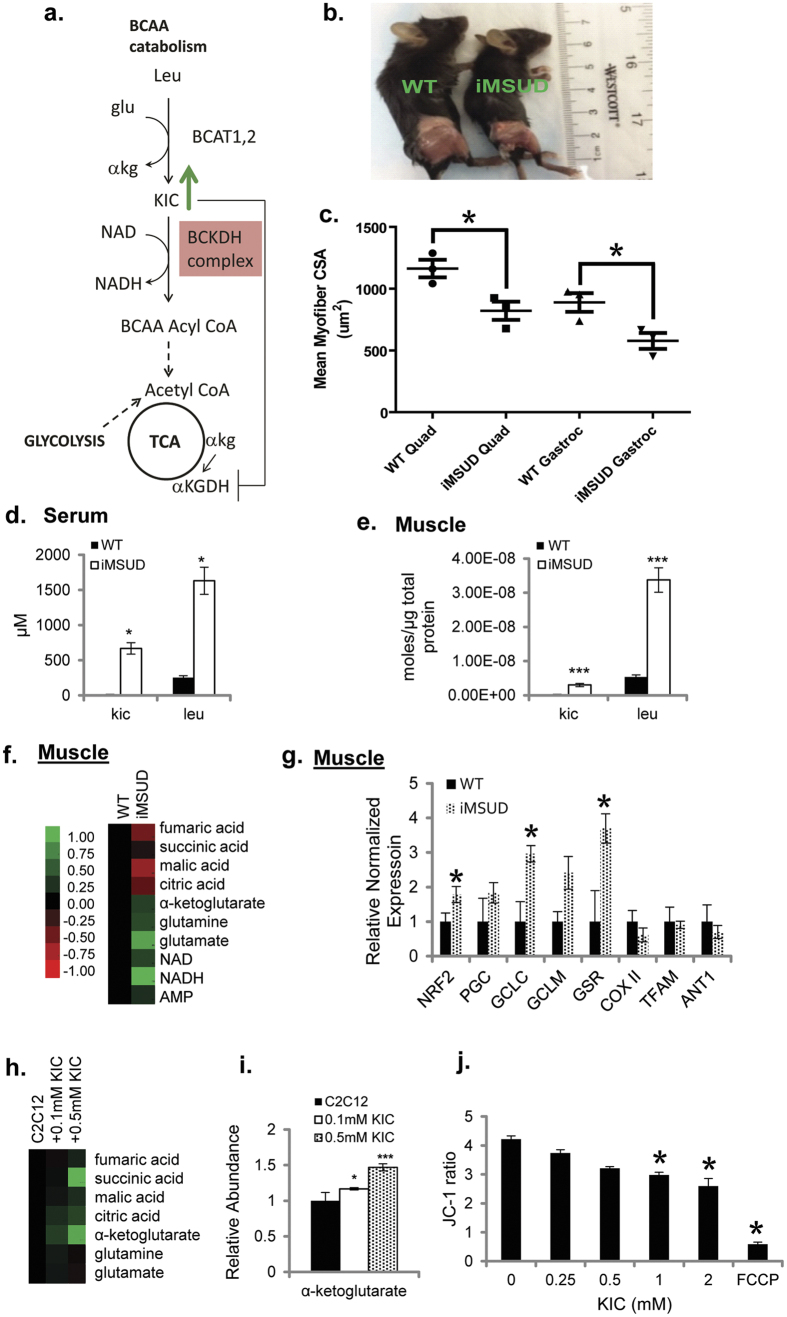
Levels of KIC and mitochondrial markers in MSUD (**a**) BCAA catabolic pathway (**b**) Image of skeletal muscle from WT and iMSUD mice. (**c**) Analysis of mean fiber cross-sectional area in quadriceps and gastrocnemius muscles. *P* values were assessed by unpaired t-test with Welch’s correction (*p ≤ 0.05). Results are shown as mean ± SEM of independent animals (n = 3). (**d**) Concentration of KIC and leucine measured in iMSUD and WT mice serum. Results are shown as mean ± SEM of independent animals (n ≥ 4). Statistical significance was determined using unpaired two-tailed Students’ t-test and is denoted by *p ≤ 0.05. (**e**) Concentration of KIC and leucine measured in iMSUD and WT mice muscle. Results are shown as mean ± SEM of independent animals (n ≥ 3). Statistical significance was determined using unpaired two-tailed Students’ t-test and is denoted by ***p ≤ 0.001. (**f**) LC-MS based metabolic profiling of mitochondrial metabolites observed in WT and iMSUD mice. Heat map includes relative values baselined to wild-type of independent animals (n ≥ 3). (**g**) NRF2, PGC, GCLC, GCLC, GSR, TFAM, COXII, and ANT1 gene expression in WT and iMSUD mice. Indicated mRNA levels were determined by qPCR. Bar graphs indicate mean ± SEM of independent animals (n ≥ 4). *p ≤ 0.05 (**h**) LC-MS based metabolic profiling of mitochondrial metabolites measured in C2C12 skeletal muscle cells treated with KIC (0.1–0.5 mM) for 48 h. Heat map includes relative values baselined to untreated C2C12 skeletal muscle cells of 4 independent preparations.(**i**) Extracellular *α*-ketoglutarate secreted by the C2C12 muscle cells treated with KIC (0.1–0.5 mM) for 48 h. Bar graphs indicate mean ± RSD of 4 independent preparations. Statistical significance was determined using unpaired two-tailed Students’ t-test and is denoted by ***p ≤ 0.001 and *p ≤ 0.05. (**j**) JC-1 fluorescence ratio of C2C12 skeletal muscle cells treated with KIC (0.25–01.0 mM) for 48 h. Data indicate mean ± SD of 4 independent preparations.

As skeletal muscle is the major site of amino acid catabolism and a source of BCKA, it is not surprising that human patients with MSUD display muscle fiber abnormalities, specifically altered fiber diameter. Upon analysis of gross anatomy, we observed that skeletal muscle mass in iMSUD mice was reduced compared to wild-type littermate controls (Fig. 1b). To determine whether the iMSUD mouse model had muscle fiber defects similar to that observed in patients with MSUD, quadriceps and gastrocnemius muscles were isolated from iMSUD mice and compared to wild-type littermate controls. iMSUD mice displayed a significant reduction in muscle fiber size, suggesting muscle atrophy (Fig. 1c).

### iMSUD mice display elevated KIC and mitochondrial dysfunction

KIC and its’ corresponding branched-chain amino acid (BCAA) precursor, leucine are the major toxic metabolites associated with MSUD related symptoms^20^, therefore we focused on KIC metabolism in this study. Using LC-MS based metabolomics, we measured changes in KIC and leucine concentrations from serum and muscle of 25-day-old iMSUD mice (*Dbt*^*tm1Geh*^/*Dbt*^*tm1Geh*^; Tg (tetO-DBT) A1Geh/J; Tg(Cebpb-tTA)5Bjd) and age-matched wild-type mice. iMSUD mice showed a significant increase in branched chain keto-acid (BCKA), KIC and leucine. Leucine was significantly increased 6.4-fold in serum (1.63 ± 0.19 mM) and 6.3-fold in muscle isolated (33.73 ± 4.38 nmoles/μg of total protein) (Fig. 1d,e) from iMSUD mice compared wild-type controls. Similarly, KIC was significantly increased 40-fold (668.05 ± 81.74 μM) in serum and 15-fold (3.02 ± 0.56 nmoles/μg of total protein) in muscle of iMSUD mice (Fig. 1d,e) compared with wild-type controls. Since the KIC levels in wild-type animals were extremely low as compared to the mutant (6.3 and 40 fold changes in leucine and KIC respectively), the graph of these levels are not visible in Fig. 1d,e. These results are consistent with MSUD-related mutations in BCKDH leading to a dramatic (up to 10 fold) buildup in BCAA and BCKAs in MSUD mouse models and patient serum^13^,^21^, brain^21^, and liver^21^.

In skeletal muscle (quadriceps), we observed a decrease in TCA intermediates and a moderate but statistically insignificant accumulation of *α*-ketoglutarate and its’ precursor glutamate (Fig. 1f,g). In the mitochondria, α-ketoglutarate is generated in the TCA (tricarboxylic acid) cycle and also by glutamate dehydrogenase during the oxidative deamination of glutamate. *α*-ketoglutarate is then oxidatively decarboxylated by the alpha-ketoglutarate dehydrogenase complex (*α*-KGDH) to form succinyl-CoA. Overall this suggests that in MSUD, the buildup of KIC inhibits the TCA enzyme *α*-KGDH^22^, leading to the accumulation of alpha-ketoglutarate and reduced TCA Cycle flux observed in the form of a decrease in TCA intermediates. In agreement with these findings, previous studies have shown KIC-dependent inhibition of mitochondrial metabolism using isolated rat liver cells^23^,^24^,^25^ and specifically of the enzyme *α*-KGDH using *in vitro* studies^22^,^26^. Additionally, our results revealed an increase in AMP and NADH levels in iMSUD mice, suggesting ATP depletion, a hallmark of mitochondrial dysfunction. To further determine if mitochondrial function is altered in MSUD, we examined the expression of a subset of genes involved in mitochondrial metabolism and biogenesis^27^ in muscle of iMSUD mice and wild-type mice using real-time quantitative PCR (RT-qPCR). mRNA levels (Fig. 1g) of mitochondrial genes (cytochrome c oxidase subunit II (COX II), adenine nucleotide translocase-1 (ANT1), and mitochondrial transcription factor A (TFAM)) were significantly decreased in iMSUD mice compared to wild-type.

It is well-known that pathways related to oxidative stress-response are activated by mitochondrial dysfunction in skeletal muscle^28^. Likewise, free radicals and oxidative stress have been linked to *α*-KGDH inhibition in various neurological disorders^29^,^30^,^31^ and implicated in the pathogenesis of MSUD^32^,^33^,^34^. In agreement, NRF2 (a key transcription factor that senses and mediates responses to redox stress^35^) and its targets were significantly increased (Fig. 1g). In conjunction with this, we did not observe any changes in GSH/GSSG levels (ratios of reduced to oxidized glutathione which correlate with oxidative stress) in iMSUD mice compared to controls (Figure S1). This suggests that the up regulation of the NRF2 pathway may help to maintain oxidative homeostasis in spite of mitochondrial dysfunction. Together, these results suggest that in MSUD the accumulation of KIC and its effects on mitochondria are associated with deleterious effects on metabolic homeostasis.

### Treatment with disease relevant levels of KIC causes accumulation of *α*-ketoglutarate and mitochondrial dysfunction *in vitro*

Since the concentration of KIC in the serum of iMSUD mice was ~0.5 mM, we tested whether chronic treatment with disease relevant levels of KIC was sufficient to recapitulate mitochondrial dysfunction in skeletal muscle cells. C2C12 skeletal muscle cells treated with concentrations of KIC (0.1–0.5 mM, derived from Fig. 1d) caused a statistically significant increase in alpha-ketoglutarate (Fig. 1i), which is consistent with the increase in α-ketoglutarate observed in iMSUD mice (Fig. 1f,j). To evaluate whether the buildup of KIC can lead to changes in mitochondrial metabolism, the mitochondrial transmembrane potential (ψ) was assessed by JC-1 fluorescence. A decrease in the JC-1 fluorescence intensity ratio was observed in C2C12 skeletal muscle cells treated with KIC for 48 hours (Fig. 1k), suggesting an inhibition of mitochondrial metabolism. This is in agreement with previous studies where KIC was shown to cause changes in mitochondrial membrane potential^36^,^37^. Our data further reinforce the hypothesis that the toxic accumulation of the metabolite KIC is a primary cause of mitochondrial dysfunction in MSUD. It is possible that increased leucine levels also contribute to pathogenesis independent of conversion to KIC. But it is interesting that the knock-out of BCAT2 (the enzyme that produces KIC) does not result in MSUD-like phenotypes and in fact the mice are healthy and lean^38^. Together, our results demonstrate that the accumulation of KIC associated with MSUD can cause mitochondrial dysfunction suggesting that drugs that modulate leucine metabolism and accumulation of KIC will be beneficial in treatment of MSUD.

### LC-MS based chemical screening of MSUD patient derived fibroblasts reveals that Metformin diminishes the accumulation of KIC

Currently there are no FDA-approved drug therapies for the long-term management of MSUD, although a small number of patients with a specific mutation in the thiamine binding site in the E1b subunit of BCKDH (30–40% activity), are known to respond to thiamine supplementation^11^. It has also been shown that a subset of patients can respond to phenybutyrate which has been shown to inhibit the ability of the kinase BCKDK to bind to and inhibit the enzyme BCKDH^39^. Here, we present an alternate drug screening strategy for reprogramming MSUD metabolism and decreasing the accumulation of KIC.

**Figure 2:**
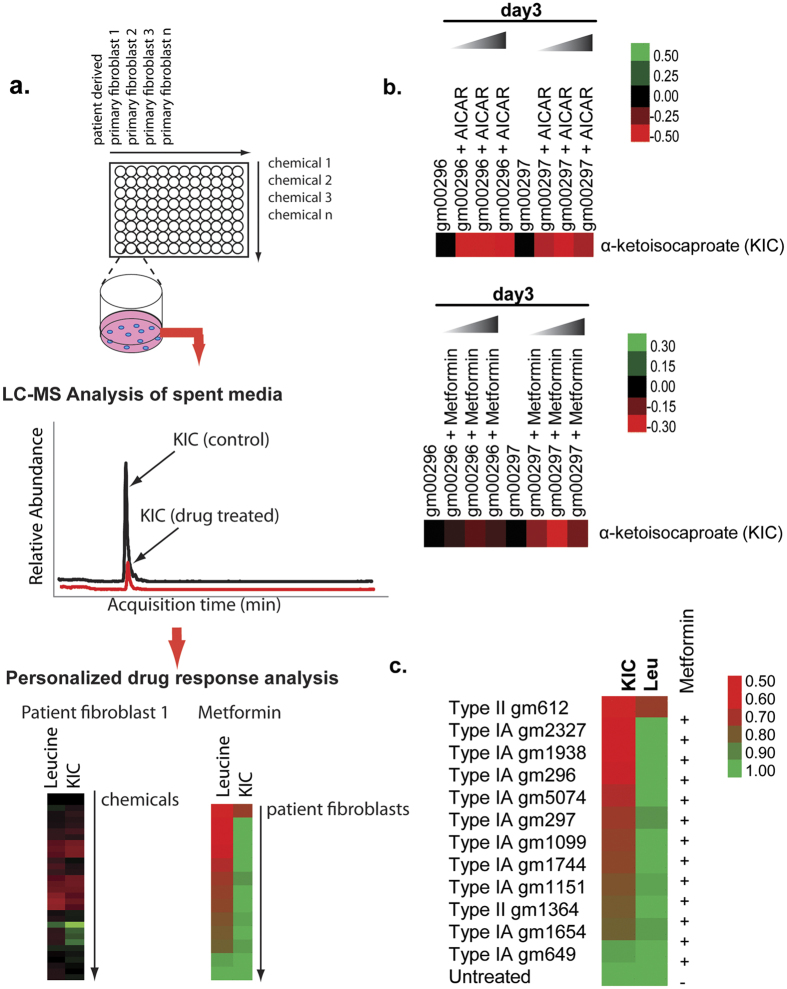
LC-MS based chemical screening of MSUD patient derived fibroblast reveals that Metformin diminishes the accumulation of KIC and induces a glycolytic switch (**a**) 96-well plate LC-MS based assay for screening drugs that modulate leucine metabolism in patient cells. MSUD fibroblasts were cultured in a 96-well plate and treated with an in-house curated collection of modulators of pathways in central carbon metabolism-AMPK, mTOR, fatty acid oxidation and oxidative phosphorylation. Following 72 h treatment, spent media was collected and diluted 10-fold for LC-MS analysis to measure levels of leucine and KIC in MSUD fibroblasts. (**b**) Extracellular KIC measured in patient derived MSUD cells (gm297, gm296) treated with Metformin (0.25–1 mM) and AICAR (25–100 μM) for 72 h. Heat map includes relative values baselined to untreated gm296 and gm297 of 2 independent preparations. (**c**) Extracellular KIC and leucine measured in Type IA and II patient derived MSUD cells treated with Metformin (0.5 mM) for 72 h. Heat map includes relative values baselined to untreated of 3 independent preparations.

We developed a 96-well plate LC-MS based assay for screening drugs that modulate leucine metabolism in patient cells (Fig. 2a). Type IA MSUD fibroblasts purchased from Coriell Inc. were cultured as recommended and treated with drugs for 72 hours. The drugs screened were an in-house curated collection of modulators of pathways in central carbon metabolism-AMPK, mTOR, fatty acid oxidation and oxidative phosphorylation. Following treatment, spent media was collected and diluted 10-fold for LC-MS analysis to measure elevated levels of leucine and KIC. From the screen we discovered that treatment with AMP kinase (AMPK) activators AICAR and Metformin decreased the accumulation of extracellular KIC (Fig. 2b). AICAR achieved its maximal inhibition at 50 μM and did not show increased inhibition of KIC levels at higher concentrations. On the other hand, metformin showed a concentration dependent maximal decrease in KIC levels at 0.5 mM. Interestingly at 1 mM it was less effective at decreasing levels of KIC suggesting off-target effects. Though both drugs can activate the AMPK energy stress response pathway, it has recently been shown that metformin might do so by directly targeting the mitochondrial redox shuttle enzyme, glycerolphosphate dehydrogenase and alter the redox state of the mitochondria^40^,^41^.

We decided to investigate the therapeutic potential of Metformin rather than AICAR, since, Metformin is a largely safe, FDA approved, and widely prescribed anti-diabetic drug^42^. Therefore, a therapeutic effect of this drug in cellular and mouse models of MSUD can result in a more rapid translational study in human patients. Physiologically, Metformin is well characterized to lower glucose levels by improving glucose uptake in peripheral tissues such as the muscle and also reducing glucose production in the liver^43^,^44^. Mechanistically, this biguanidine drug has been shown to have at least two targets, activating AMPK and inhibiting complex I of the electron transport chain^45^,^46^. In secondary assays, we observed that levels of KIC decreased with Metformin treatment (0.5 mM) over the course of 72 h. The most significant decrease in KIC was observed at 48 and 72 h (Figure S2A). At this time point, we observed a slight increase in extracellular leucine, suggesting a concordant decrease in leucine catabolism (Figure S2A). To investigate whether the effects of Metformin on KIC accumulation would be patient dependent, we measured the effects of Metformin on a comprehensive array of patient derived fibroblasts obtained from Coriell Inc., representing both Type 1A and II patients. After treating with Metformin (0.5 mM) for 72 h, the accumulation of KIC decreased in 9 out of 10 lines compared to untreated (Fig. 2c). We did not see significant changes in leucine levels upon Metformin treatment (Fig. 2c). Treatment with Metformin resulted in significant decreases in genes associated with BCAA catabolism at 72 hours of treatment (Figure S2B) in a patient derived fibroblast. In conjunction, an increase in glycolytic enzyme hexokinase 2 (HK2) and lactate production was observed in response to Metformin (Figure S2C,D). This suggests that Metformin inhibits the catabolism of leucine and promotes glycolytic metabolism as a source of carbon in MSUD cells.

### Metformin inhibits KIC accumulation and promotes metabolic homeostasis in iMSUD mice

**Figure 3:**
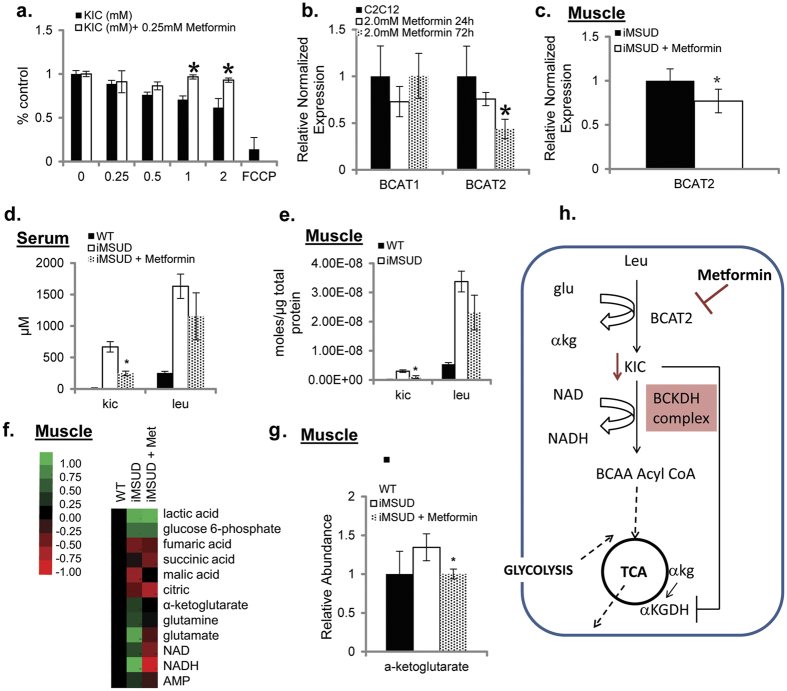
Metformin inhibits BCAA catabolism and promotes metabolic homeostasis in iMSUD mice (**a**) JC-1 fluorescence ratio of C2C12 skeletal muscle cells treated with KIC (0.25–01.0 mM) ±Metformin (0.25 mM) for 48 h. Data indicate mean ± SD of 4 independent preparations. *p ≤ 0.05 (**b**) BCAT1 and BCAT2 gene expression of C2C12 muscle cells treated with Metformin (2.0 mM) for 48–72 h. Indicated mRNA levels were determined by qPCR. Data indicate mean ± SEM. *p ≤ 0.05 (**c**) BCAT2 gene expression WT and iMSUD mice fed a standard 5LG6 diet (Lab Diet) or 5LG6 with 0.1% (w/w) Metformin *ad libitum.* Indicated mRNA levels were determined by qPCR. Bar graphs indicate mean ± SEM of independent animals (n ≥ 4). Statistical significance was determined using unpaired two-tailed Students’ t-test and is denoted by *p ≤ 0.05. LC-MS profiling of BCAA metabolites measured in (**d**) skeletal muscle and (**e**) serum from WT and iMSUD fed a standard 5LG6 diet (Lab Diet) or 5LG6 with 0.1% (w/w) Metformin *ad libitum*. Results are shown as mean ± SEM of independent animals (n ≥ 3). Statistical significance was determined using unpaired two-tailed Students’ t-test and is denoted by *p ≤ 0.05. (**f**) LC-MS profiling of mitochondrial metabolites measured in skeletal muscle from WT and iMSUD mice fed a standard 5LG6 diet (Lab Diet) or 5LG6 with 0.1% (w/w) Metformin *ad libitum*. Heat map includes relative values baselined to WT of independent animals (n ≥ 3). (**g**) α-ketoglutarate measured in skeletal muscle from WT and iMSUD mice fed a standard 5LG6 diet (Lab Diet) or 5LG6 with 0.1% (w/w) Metformin *ad libitum.* Results are shown as mean ± SEM of independent animals (*n* ≥ 3). Statistical significance was determined using unpaired two-tailed Students’ t-test and is denoted by *p ≤ 0.05. (**h**) Mechanism of Metformin-induced inhibition of the BCAA pathway via mitochondrial BCAT2.

We next examined whether this metabolic reprogramming by Metformin could promote metabolic homeostasis in a physiological context using the iMSUD mouse-model. Testing if Metformin can inhibit KIC and leucine accumulation in serum and skeletal muscle, and alleviate mitochondrial dysfunction in skeletal muscle. First, the effects of Metformin were studied using an *in vitro* model of skeletal muscle myotubes differentiated from C2C12 myoblasts. Treatment with 0.25 mM Metformin was able to rescue the inhibitory effects of KIC treatment on mitochondrial potential (Fig. 3a). In order to investigate the mechanism by which Metformin could inhibit BCAA catabolism and KIC accumulation, we measured transcriptional levels of the branched-chain amino acid transaminases (BCAT) 1 and 2. The enzymes BCAT-1 and 2 are directly upstream of BCKDH (the enzyme mutated in MSUD) and catalyze the reversible transamination of leucine to KIC, (Fig. 1a). Metformin treatment at 24 and 72 hours showed no change in the expression of BCAT-1 (cytoplasmic isoform) but revealed a significant inhibition in the expression of BCAT-2 (mitochondrial isoform) (Fig. 3b). These findings suggest a mitochondria-specific role for Metformin in modulating BCAA catabolism. This effect was conserved *in vivo*, as judged by measuring the expression of skeletal muscle BCAT-1 and 2 in iMSUD mice treated with Metformin for 4 days (Figs 3c and S3).

In order to compare relevant Metformin doses used in *in vivo* (iMSUD skeletal muscle) and *in vitro* (patient fibroblasts) experiments, we normalized amounts of Metformin to total protein. We measured the intra-cellular concentrations of Metformin that resulted from 0.5 mM drug treatment of Type IA MSUD fibroblasts (gm297, gm296), finding the level to be 0.14 nmoles per microgram. Treatment of mice with 0.1% w/w Metformin chow also resulted in similar concentrations of the drug in skeletal muscle (0.1 nmole per microgram of protein), suggesting that this *in vivo* administration regime results in levels that mediate effects *in vitro*. In skeletal muscle, mRNA levels of mitochondrial BCAT-2 (Fig. 3c) but not cytoplasmic BCAT-1 (Figure S4) were significantly decreased in mice treated with Metformin, suggesting that BCAT-2 is a relevant target of Metformin both *in vitro* and *in vivo*. Inhibition of BCAT-2 can reduce mitochondrial levels of KIC, thereby alleviating inhibition of mitochondrial enzyme *α*-KGDH. In agreement, treatment of iMSUD mice with 0.1% w/w Metformin chow for 4 days inhibited the intramuscular accumulation of both KIC and leucine (Fig. 3d) suggesting that Metformin inhibits leucine catabolism in muscle. In parallel, we also observed a decrease of KIC and leucine accumulation in serum from iMSUD mice (Fig. 3e). The Metformin-meditated reduction of BCAT-2 expression and KIC accumulation in iMSUD mouse muscle and serum are consistent with the BCAT-2 knock-out mouse, which accumulates BCAAs (leucine) and has decreased levels of BCKA (KIC) in serum^38^. Interestingly, these mice are remarkably healthy, lean, and resistant to diet induced obesity and insulin resistance.

As described previously, iMSUD mice show an intra-muscular accumulation of AMP and NADH, and *α*-ketoglutarate suggesting metabolic dysfunction. Increased AMP and NADH suggest inhibition of mitochondrial ATP synthesis via oxidative phosphorylation. Metabolic profiling of skeletal muscle from iMSUD mice fed 0.1% w/w Metformin chow for 5 days shows that the Metformin is able to reverse the levels of AMP, NAD, NADH, glutamate, and *α*-ketoglutarate (Fig. 3f,g), consistent with restored mitochondrial function.

Overall (Fig. 3h), our work indicates therapeutic benefits of Metformin-treatment in MSUD by 1) decreased mitochondrial accumulation of leucine and KIC and 2) restoration of mitochondrial metabolism. The wide-spread and safe use of Metformin for treating diabetes, suggests that translational studies can be readily carried out for repurposing this drug for long-term management of MSUD in conjunction with currently used dietary interventions.

## Discussion

Dysregulation of Branched Chain Amino Acid (BCAA) metabolism is associated with a range of diseases including chronic conditions such as type II diabetes, as well as severe childhood disorders. Of this spectrum of diseases, the Maple Syrup Urine Disease (MSUD) is an orphan disease and inborn error in metabolism that arises from mutations in the E1 or E2 subunits of the BCKDH enzyme. This enzyme is first irreversible step in BCAA catabolism and its dysfunction leads to the incomplete metabolism of BCAAs, resulting in the accumulation of keto-acid intermediates in blood. Animal models are well suited to explore the pathophysiology of MSUD. They also present opportunities for pre-clinical testing of drug interventions. Homniacs *et al.* generated a classical MSUD (cMSUD) model by knocking out the E2 subunit of BCKDH^6^. Similar to human patients who fail to receive treatment, cMSUD mice die within a few days of birth. The extremely short lifespan of this model makes it challenging to study therapeutic interventions, unless prenatal treatments are desired. In this study, we used the intermediate MSUD (iMSUD) model also generated by Homniacs *et al.*^5^,^6^. Using this biological model, we show that iMSUD mice have skeletal muscle atrophy associated with biochemical dysfunction (Fig. 1), Similarly MSUD patients also display skeletal muscle dysfunction and diminishment in fiber diameters, which interestingly and does not directly correlate with leucine levels in the blood^12^. Here we suggest that accumulation of keto-acids is the likely the proximal cause of toxicity in skeletal muscle, and that decreasing the intracellular accumulation of KIC and leucine can help to restore metabolic homeostasis.

Currently in order to manage this disease, patients are kept under lifelong clinical management with a diet reduced in BCAAs. BCAAs are essential amino acids and important in protein synthesis and regulation of nutrient-sensitive signaling pathways e.g. mTOR^47^. Therefore, alternative or complementary approaches to managing this disease will be of therapeutic importance. Small molecules that can help restore metabolic homeostasis and muscle function will provide enormous benefits, as well as provide a better understanding of the regulation of BCAA catabolism. Using a novel LC-MS based platform and patient derived fibroblasts (Fig. 2) we show that the FDA-approved drug Metformin reduced KIC accumulation. It has now been shown that metformin non-competitively inhibits the redox shuttle enzyme glycerophosphate dehydrogenase^40^,^41^, which transports glycolysis generated NADH as a source of electrons for the mitochondrial electron transport chain. This inhibition alters the redox state of the mitochondria. This suggests that metformin can alter the mitochondrial redox state and there by regulate the activity of mitochondria localized BCAA catabolic enzymes including BCAT and BCKDH. This may explain the connection between metformin and BCAA catabolism. This effect of metformin in reducing levels of KIC is conserved in skeletal muscle cells *in vitro* and *in vivo*, and is associated with a decrease in expression of the mitochondrial isoform of mitochondrial BCAT (Fig. 3). BCAT is a mitochondrial transaminase enzyme that converts leucine to KIC. KIC, at disease relevant concentrations can inhibit mitochondrial function (Fig. 3) by inhibiting mitochondrial alpha-ketoglutarate dehydrogenase^22^. This suggests that Metformin may have a therapeutic effect by diminishing the mitochondrial accumulation of KIC in skeletal muscle cells, and decreasing the KIC mediated inhibition of mitochondrial metabolism. BCAT is increasingly recognized as a key regulator of physiology^38^, and this study points to a new novel target of Metformin in the MSUD disease context. The ability of Metformin to restore metabolic homeostasis in these models would suggest that this drug could be used for long-term metabolic reprogramming and management of MSUD in patients, especially those with intermediate and intermittent MSUD where there is residual activity of the BCKDH enzyme. It is unclear if classic MSUD will be impacted by this drug though the ability to reduce levels of mitochondrial KIC could still be relevant for promoting mitochondrial metabolism. It is unclear if Metformin mediated decrease in buildup of mitochondrial KIC will be sufficient to restore skeletal muscle homeostasis in MSUD. Ongoing studies will determine if long-term treatment with metformin can significantly reduce muscle atrophy and increase life-span of mutant animals.

## Materials and Methods

### Chemicals

All chemicals and metabolite standards were purchased from Sigma (St Louis, MO) unless otherwise specified. LC-MS grade solvents acetonitrile and methanol were purchased from VWR (Radnor, PA). The Leucine-1-^13^C was purchased from Santa Cruz Biotechnology (Dallas, TX). Deionized water was generated in-house for mobile phase preparation. The Vivaspin500 centrifugal concentrator devices (5,000 MWCO PES) were purchased from Vivaproducts (Littleton, MA).

### Animals

The iMSUD mouse model used in these studies was described as the “A” line by Homanics *et al.*^5^,^6^. iMSUD and wild-type control mice were maintained on a mixed background by breeding *Dbt*^*tm1Geh*/+^; Tg(tetO-DBT)A1Geh/J; Tg(Cebpb-tTA)5Bjd) mice to generate *iMSUD* or *Dbt1*^+/+^ mice homozygous for the LAP-tTA and TRE-E2 transgenes. Breeders were fed a PicoLab Mouse Diet 20 (5058, Lab Diet (St. Louis, MO, USA) and at 21 days of age progeny were weaned and fed a standard 5LG6 diet (Lab Diet) or 5LG6 with 0.1% (w/w) Metformin *ad libitum*. Pure Metformin was obtained from Sigma (St. Louis, MO, USA) and mixed to homogeneity during manufacturing of the diets (Lab Diet). After 4 days of treatment serum and tissue were isolated. Tissue was frozen in liquid nitrogen, stored at −80 °C, and cryohomogenized prior to processing for analysis. All animal care and experimental procedures were approved by the Institutional Animal Care and Use Committee at the Buck Institute for Research on Aging. The work was carried out in accordance with the approved guidelines.

### Myofiber CSA

Tissue samples were fixed in 4% paraformaldehyde solution in PBS before being embedded in paraffin. Morphology was examined in the hematoxylin and eosin-stained section. The size of muscle fibers was quantified in Aperio ImageScope.

### Cell Culture

Type IA and II MSUD fibroblasts (purchased from Coriell Inc., Camden, NJ, USA) (cat#GM00297, GM00296, GM10744, GM02327, GM01938, GM05074, GM11151, GM01654, GM00612, GM01364, GM01099, GM00649) were maintained in Eagle’s Minimum Essential Medium (Invitrogen, Carlsbad, CA, USA) with Earle’s salts and non-essential amino acids supplemented with 15% (v/v) fetal bovine serum (Invitrogen), glutamine (2 mM; Invitrogen) and penicillin/streptomycin (100 units/mL/100 μg/mL; Invitrogen) at 37 °C in 5% CO_2_. Cells at early passages (between 18 and 25 passages) were used in all experiments.

### PCR

Total RNA was extracted from cells and tissue using the Quick-RNA MiniPrep kit (Zymo Research, Irvine, CA, USA) according to the manufacturer’s recommended instructions. RNA samples were then quantified by a NanoDrop 2000 (Thermo Scientific, Waltham, MA, USA). cDNA was synthesized from 0.25–0.5 μg of total RNA template using the iScript 5x RT Supermix (Bio-Rad, Hercules, CA, USA). Reactions occurred in a T100 Thermal Cycler (Bio-Rad) according to the manufacturer’s recommended instructions. Real-Time Quantitative PCR analysis was performed using a Bio-Rad CFX Connect Real-Time PCR Detection System. Acquisition of data was then performed using Bio-Rad CFX Manager software. Each PCR reaction comprised of both forward and reverse primers at a concentration of 400 nM with 1 μL of cDNA diluted five-fold, 7.4 μL of nuclease-free water, and 10 μL of Sso Advanced Universal SYBR Green Supermix (Bio-Rad). Sample data values from quantitative PCR analysis were normalized to Actin or TATA Box binding Protein, TBP. Primer sequences are listed in Supplemental Experimental Procedures.

### Measurement of mitochondrial membrane potential (ψ) *in vitro*

C2C12 mouse skeletal muscle cells were seeded at 2,000 cells per well in a 96-well dish. Cells were treated for 48 hours with 0.25, 0.5, and 1 mM KIC and the assay was conducted following the JC-1 Mitochondrial Membrane Potential Assay Kit protocol (Cayman Chemical Company, Ann Arbor, MI, USA), with FCCP as the cell death control marker. Mean fluorescence was then measured using a plate reader with excitation and emission at 535 nm and 595 nm, respectively.

### Metabolite Extraction of Serum

5 μL of serum was diluted in 20 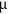
L PBS. A volume of 25 uL of 50% methanol +2 μg/mL c13 leucine (internal standard) and 75 μL chloroform were added to serum and mixed for 1 min for metabolite extraction. The samples were stored overnight at −20 °C. Following overnight incubation, the sample was centrifuged at 10,000 g for 15 min at 4 °C to separate the organic and aqueous layers. The 30 μL aqueous layer was diluted with 170 uL 50% methanol and then transferred to a Vivaspin500 centrifugal concentrator unit with an YM-3membrane (3 kDa molecular weight cutoff (MWCO PES)). The solution was spun down at 12,000 g for 75 min at 4 °C and the flow-through was recovered and analyzed by LC-MS. It should be noted that the membrane was spin-rinsed with deionized water 3 times to remove trace amounts of glycerin before applying the sample.

### Metabolite Extraction of Muscle

Approximately 25 mg of cryohomogenized frozen tissue powder were sonicated with 100 μL 50% methanol +2 μg/mL c13 leucine (internal standard) on ice. A volume of 300 μL of chloroform was added to each sample and mixed for 1 min for metabolite extraction. The samples were stored overnight at −20 °C. Following overnight incubation, the sample was centrifuged at 10,000 g for 15 min at 4 °C to separate the organic and aqueous layers. 75 μL of the aqueous layer was recovered and analyzed by LC-MS.

### LC-QTRAP-MS

For quantitative LC-MS analysis, HPLC was performed using a Shimadzu UFLC prominence system fitted with following modules: CBM-20A (Communication bus module), DGU-A_3_ (degasser), two LC-20AD (liquid chromatography, binary pump), SIL-20AC HT (auto sampler) and connected to a Phenomenex Luna NH_2_ (2.0 mm × 150 mm, 3.0 μM) column. The solvent system was A = 20 mM ammonium acetate pH 9.5 with 5% acetonitrile and B = acetonitrile. The starting gradient conditions were 95% B at a flow rate of 0.3 mL/min. The following gradient program was used: 0 to 20 min, 95-10%B, 25–30 min 10%B, and 30.1–35 min 95%B. Samples were kept at +4 °C, and the injection volume was 10 μL.

Mass spectrometric analysis was conducted using negative ion electrospray ionization in the multiple reaction monitoring mode (MRM) or multiple ion mode (MI) on an API 4000 QTRAP (Foster City, CA, USA) mass spectrometer fitted with a TurboV^TM^ ion source. The ionization parameters were set as follows: curtain gas (CAD); 20psi; collision gas: medium; ion spray voltage (IS): −4500 V; Temperature (TEM): 550 °C; Ion source Gas 1 (GS1); 60psi; and Ion source Gas 2 (GS2): 50psi. The compound-specific parameters were established using the appropriate standards (Table S2).

AB SCiex’s Analyst^®^v1.6.1 was used for all forms of data acquisition and method development. AB SCiex’s Analyst^®^v1.6.1 was used for all forms of data acquisition and an in-depth analysis of the HPLC-MS data, specifically for calculating the peak areas for the identified features from serum, muscle, and cellular extracts. All data were presented as means +/− SEM. Comparisons between groups were performed using Student t-tests, 2-tails. P ≤ 0.05 was considered significant and p ≤ 0.005 considered highly significant (designated * and ** on bar graphs, respectively).

## Additional Information

**How to cite this article**: Sonnet, D. S. *et al.* Metformin inhibits Branched Chain Amino Acid (BCAA) derived ketoacidosis and promotes metabolic homeostasis in MSUD. *Sci. Rep.* **6**, 28775; doi: 10.1038/srep28775 (2016).

## Supplementary Material

Supplementary Information
