# Supplementary material for: Metformin inhibits Branched Chain Amino Acid (BCAA) derived ketoacidosis and promotes metabolic homeostasis in MSUD
Source: Sci Rep. 2016 Jul 4;6:28775. doi: 10.1038/srep28775 (PMC4931503; doi:10.1038/srep28775)
Supplement: Supplementary Information [file srep28775-s1.pdf]

Metformin inhibits Branched Chain Amino Acid (BCAA) derived ketoacidosis and promotes metabolic homeostasis in MSUD

Davis Sonnet S.*[1], O'Leary Monique N.*[2], Gutierrez Mark A.[3], Nguyen Steven M.[1], Mateen Samiha[1], Yuehmei Hsu[1], Mitchell Kylie P.[1], Lopez Antonio J.[4], Vockley Jerry [5], Kennedy Brian K.[1], and Ramanathan Arvind[1]**

Affiliation

[1] Buck Institute for Research on Aging, 8001 Redwood Blvd, Novato, CA 94945

[2] University of Michigan, Department of Pathology, 1150 W Medical Center Dr #7520, Ann Arbor, MI 48109

[3] University of Colorado, Denver Anschutz Medical Campus 13001 E. 17th Pl. Aurora, Colorado, 80045

[4] University of California, Davis 1 Shields Ave, Davis, CA 95616

[5] Children's Hospital of Pittsburgh of UPMC, 4401 Penn Ave. Pediatric Medical Genetics - 3rd Floor, Pittsburgh, PA 15224

* Authors contributed equally

** Correspondence to aramanathan@buckinstitute.org

SUPPLEMENTAL FIGURE & TABLE

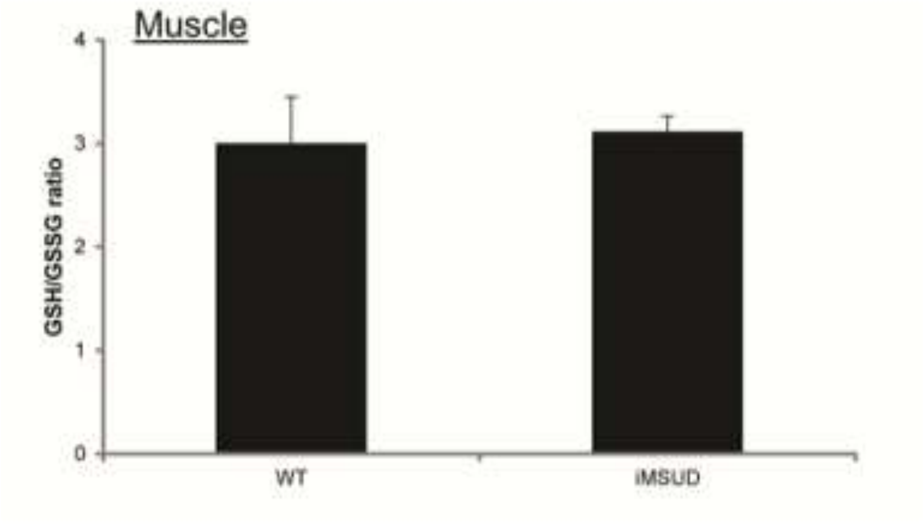

Figure S1. Intramuscular GSH/GSSG ratio measured in iMSUD and WT mice

Results are shown as mean ± SEM of independent animals (n ≥ 3).

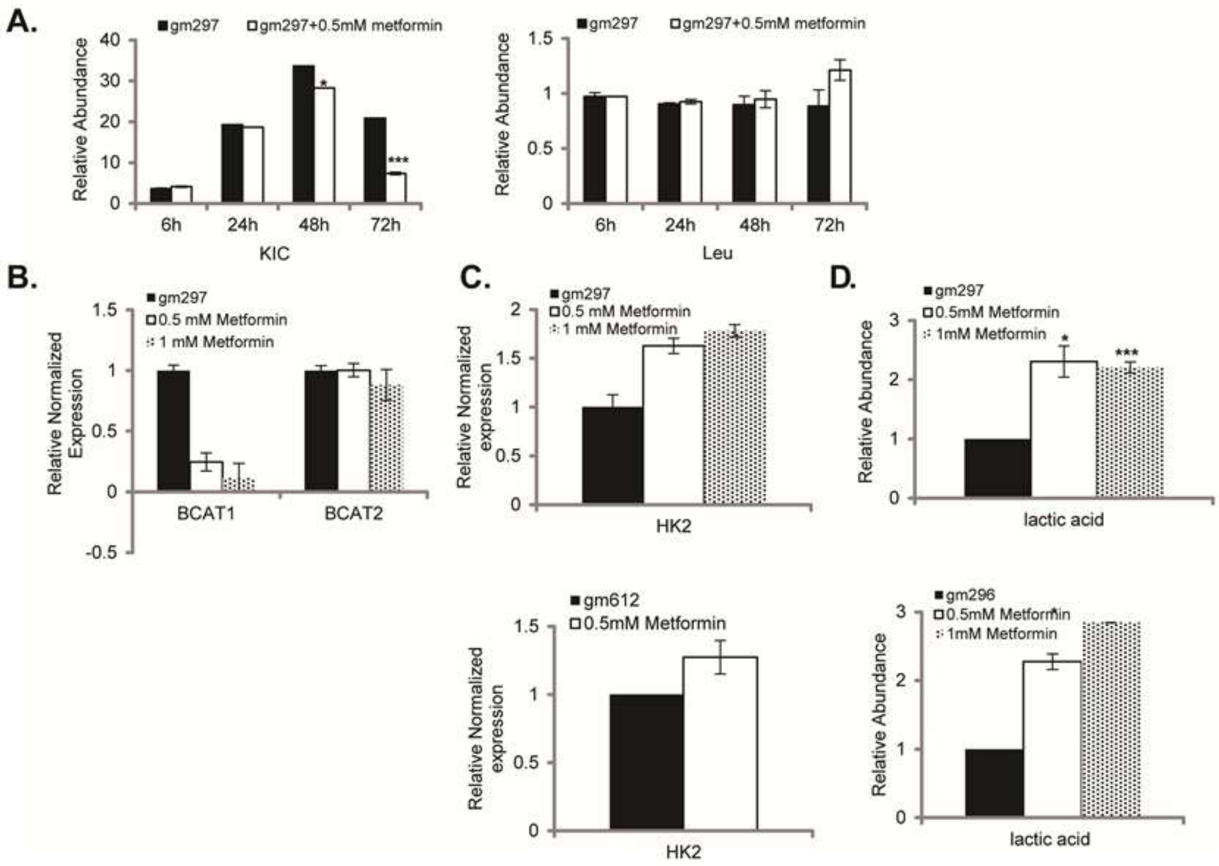

Figure S2. Metabolic effects of metformin in patient derived fibroblasts (a) Extracellular KIC and leucine measured in patient derived cells (gm279) treated with Metformin (0.5mM) for 6-72h. Values are mean ± RSD of 3 independent preparations. Statistical significance was determined using unpaired two-tailed Students' t-test and is denoted by ***p ≤ 0.001 and*p ≤ 0.05. **(b)** BCAT1and BCAT2 gene expression in patient derived MSUD cells (gm297) treated with Metformin (0.25-1mM) for 72h. Data indicate mean ± SEM of 3 independent preparations. **(c)** HK2 gene expression in patient derived MSUD cells (gm29, gm612) treated with Metformin (0.25-1mM) for 72h. Values are mean ± SEM of 3 independent preparations.**(d)**Intracellular lactic acid measured in patient derived MSUD cells (gm297, gm296) treated with Metformin (0.25-1mM) for 72h, Data indicate mean ± RSD of 3 independent preparations. Statistical

significance was determined using unpaired two-tailed Students' t-test and is denoted by ***p ≤ 0.001 and*p ≤ 0.05.

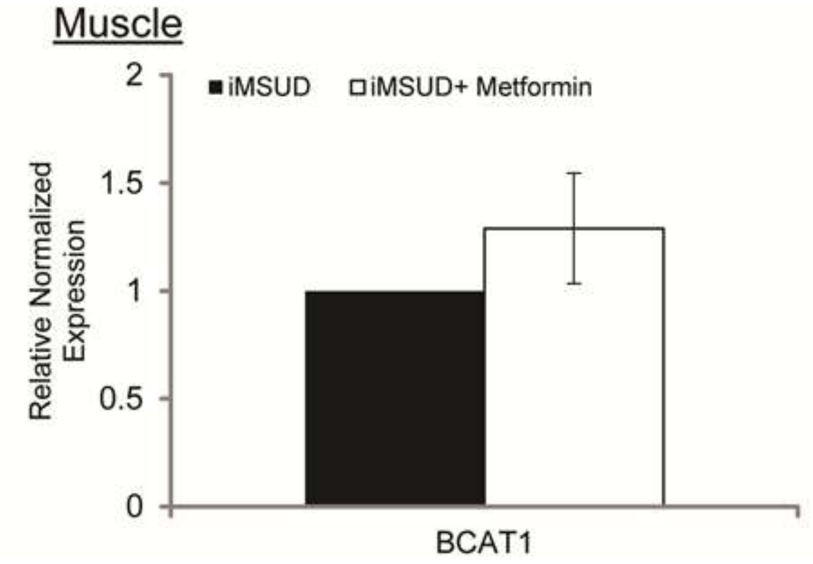

Figure S3. BCAT1 gene expression measured in skeletal muscle from iMSUD mice
Bar graphs indicate mean ± SEM of independent animals (n ≥ 4).

Gene Target	Left Sequence	Right Sequence
Tbp	CGGCTGTTTAACTTCGCTTC	CACACGCCAAGAAACAGTGA
Bcat1	GATAATGGGTGTCAGCAGGTC	GGAGGAGTTGCCAGTTCTTCT
Bcat2	AACATGGACCGGATGCTG	CCTTGTCCACTTCGATGAGC
Bckdh	CAAGGCTGGTGGGATGAG	AGTAGGTTGGGGTTGGGTTT
Hk2	GATGACTTCCGCACAGAATTT	TCTCACCCAGGTACATTCCAC
Tfam	CTGATGGGTATGGAGAAGGAGG	CCAACTTCAGCCATCTGCTCTTC
Coxii	ACGAAATCAACAACCCCGTA	GGCAGAACGACTCGGTTATC-
Ant1	GTCTCTGTCCAGGGCATCAT	ACGACGAACAGTGTCAAACG
Nrf2	CATGATGGACTTGGAGTTGC	TGCCTCCAAAGGATGTCAAT
Pgc	CCCATACACAACCGCAGTC	G-AACCCTTGGGGTCATTTG
Gclc	AGATGATAGAACACGGGAGGAG	TGATCCTAAAGCGATTGTTCTTC
Gclm	TGGAGCAGCTGTATCAGTGG	CAAAGGCAGTCAAATCTGGTG
Gsr	TTTGTGCCAACAAAGAGGAA	GAAGCCCTGCAGCATCTC

Table S1. Primers for real-time quantitative PCR. Related to Figures 1 and 3

Metabolite	Q1 (m/z)	Q3 (m/z)	Declustering Potential (DP)	Collision Energy (CE)	Entrance Potential (EP)	Cell Exit Potential CXP
leucine	132.0	86.0	+40.0	+32.0	+10.0	+7.0
leucine-1-^{13}C	133.0	86.0	+40.0	+32.0	+10.0	+7.0
KIC	129.0	n/a	-20.0	n/a	-5.0	-5.0
alanine	90.0	44.0	+30.0	+17.0	+10.0	+6.0
glutamine	147.0	130.0	+30.0	+14.0	+8.0	+8.0
glutamate	145.0	102.0	-24.0	-20.0	-5.0	-5.0
citric acid	191.0	111.0	-25.0	-19.0	-5.0	-5.0
malic acid	133.0	115.0	-25.0	-16.0	-10.0	-10.0
fumaric acid	115.0	71.0	-20.0	-12.0	-5.0	-5.0
α-ketoglutarate	145.0	57.0	-25.0	-24.0	-5.0	-8.0
succinic acid	117.0	73.0	-25.0	-17.0	-10.0	-10.0
AMP	346.1	134.0	-50.0	-47.0	-8.5	-5.0
ATP	505.9	158.9	-42.0	-40.0	-8.5	-5.0
NAD	662.0	540.0	-50.0	-25.0	-10.0	-5.0
NADH	664	79.0	-120.0	-110.0	-10.0	-5.0
lactic acid	89.0	43.0	-38.0	-17.0	-8.0	-5.0
g6p	259.0	97.0	-53.0	-18.0	-8.0	-5.0

Table S2. LC-MS compound specific parameters for multiple reaction monitoring (MRM). Related to Figures 1-3.
